# The Integrated Performance Management System: A Key to Service Trajectory Integration

**DOI:** 10.5334/ijic.5701

**Published:** 2021-11-26

**Authors:** Line Moisan, Pierre-Luc Fournier, Denis Lagacé, Sylvain Landry

**Affiliations:** 1 Interdisciplinary Chair of Research and Intervention in Health Services, Université du Québec à Trois-Rivières, 3351 Boulevard des Forges, Trois-Rivières (QC), CA; 2 Department of Information Systems and Quantitative Methods, Business School, Université de Sherbrooke, 2500 Boulevard de l’Université, Sherbrooke (QC), CA; 3 Interdisciplinary Chair of Research and Intervention in Health Services, Department of Industrial Engineering, Université du Québec à Trois-Rivières, 3351 Boulevard des Forges, Trois-Rivières (QC), CA; 4 Department of Logistics and Operations Management, HEC Montréal, 3000, chemin de la Côte-Sainte-Catherine, Montréal (QC), CA

**Keywords:** integrated performance management system, status sheet meeting, obeya, youths in difficulty

## Abstract

**Introduction:**

This article presents an experience of deploying an integrated performance management system as a catalyst for the integration of a service trajectory for children in vulnerable situations. Called ‘‘Jimmy’’, the project identifies how the integrated performance management system makes it possible to improve accessibility, continuity of services and well-being at work among stakeholders.

**Methods:**

An action research was conducted in a large healthcare organization in Canada, between August 2016 and October 2018. Data was systematically collected throughout the various cycles of research using field notes, more than 350 hours of observations, 15 interviews and 3 focus groups.

**Results:**

This research supports using an integrated performance management system as a model for collaborative management that supports both horizontal and vertical integration in the service trajectory. The use of visual boards and status sheet meetings were determining factors for service integration and the functioning of integrated teams. This also led to improvements in accessibility and continuity of services, as well as in employee well-being.

**Discussion and conclusion:**

Supported by the various tools of the integrated performance management system, Project ‘‘Jimmy’’ reinforces the implementation of linkage and coordination models, which in turn helps create strong connections among teams. The status sheet meetings and visual boards are tools that vertically integrate different hierarchical levels and horizontally integrate various front-line stakeholders through the user-oriented trajectory.

## Introduction

In recent decades, many industrialised countries have made significant reforms to their health care systems, which has led to the implementation of integration mechanisms [1]. For example, on April 1, 2015, the province of Quebec, Canada, restructured its health and social services network, going from 182 to 34 institutions, in the hopes of better integrating health care and services. Called Integrated Health and Social Services Centres (IHSSC) or Integrated University Health and Social Services Centres (IUHSSC), these new institutions now group the facilities and services for the population of a specific region. This research was conducted at one of the IHSSC.

In the wave of this change, the Quebec ministry of health and social services showed a strong desire to improve performance through the implementation of management tools. One target in its strategic planning [31] was the deployment of strategic, tactical and operational control rooms (conceptual meeting places for integrated team performance discussions) to support the initial steps of an integrated performance management system in every organisation.

“By March 31, 2018, all institutions will have deployed their strategic control room and on March 31, 2020, organisations must have deployed 80% of their tactical and operational control rooms.”

This article aims to ascertain how service integration processes are improved through the adoption of an integrated performance management system. This action research was carried out in IHSSC and specifically in the centre’s programmes for children, youth and families. In the 2015 reform, this IHSSC was created through the merger of 7 institutions that included 49 facilities spread out over more than 20,000 km^2^. This territory has experienced significant population decline and low population density, which are sources of concern when it comes to service continuity for children, youth and families. Like many regions in Quebec, the territory served by the IHSSC studied here is experiencing a demographic decline and has a low population density. These appear to be contributing factors in the review of how services are organized in their current form. This territory also has challenges when it comes to updating programs or services for youth. The insufficient pool of target clients combined with the risks of social stigma associated with marginalized groups may constitute barriers to implementing programs for family clients.

This action research, which took place over more than two years, contributes to the literature by demonstrating the operational strength of the tools of the integrated performance management system (IPMS) to answer the following question: “Which integrated performance system tools support the integration of social services for children and their families?” The literature is scant on which integrated performance management system tools foster the integration of services for a target clientele. This article aims to fill this gap.

## Conceptual Framework

### Service integration

Services can be integrated either vertically or horizontally [2,3,4,38]. Horizontal integration aligns similar services across different territories [5,6], while vertical integration aligns services that are upstream or downstream from a trajectory of care or services or found along a continuum of services [5,6]. Performance is therefore built through a trajectory of services based on a logic of complementarity between vertical and horizontal integration [6,7] and offers enormous potential to improve performance through the sharing of information that aligns the user’s trajectory [10,11].

Leutz [8] places the different types of integration along a continuum of interdependence where three levels of integration intertwine —linkage, coordination and full integration—in addition to a fourth dimension, i.e., the absence of integration [9].

Although the tools of the integrated performance management system (particularly the control rooms) have been deployed in Quebec, few studies significantly demonstrate how these rooms add value to integrating care and services for a target clientele. This paper addresses this shortcoming by exploring the integrated performance management system tools that support better vertical and horizontal integration, particularly in programs for children, youth and families.

### The integrated performance management system and its tools

The integrated performance management system currently deployed in Quebec’s health and social services network may be considered a key to the integration of services. This system is supported by a management philosophy based on continuous improvement and consists of a coherent set of standardised and coordinated principles, practices and tools that, through shared leadership, help an institution achieve clearly stated organisational objectives that aim to create value for all stakeholders.

Organizational performance requires a performance management model that establishes a basis for organizational governance and a framework for how managers should make decisions when it comes to creating and distributing value in the organization [22]. A leading reason why organizations have trouble meeting performance targets is the tendency of many managers to assess performance based on costs and efficiency. However, for an organization to achieve balance, performance needs to be measured using more criteria. In the end, the extant literature reveals that organizations adopting PMSs reach higher performance levels compared to those who do not [41].

To that end, performance assessment needs to consider multiple criteria necessary for the organization to find balance. Central to this notion is the quadruple aim [42] to assess the multidimensionality and systemic nature of a healthcare organization. This quadruple aim includes enhancing the patient experience, improving population health of a given territory, reducing costs, and improving work-life for all staff.

The tools and practices used for this action research were kaikaku, A3 reports, kaizen, control rooms, status sheets and kata.

### Tools used

Kaikaku is a Japanese word that translates as “radical change” and is seen as a process that can generate significant results when existing practices are replaced with new ones [28]. Kaikaku is usually initiated by an organisation’s strategic leadership.

The A3 report is a summary document that conveys information about projects on a single A3-sized sheet (11 × 17 inches format in North America) on which a problem-solving approach is expressed in a structured way [15]. There is a multitude of A3 reports, but two types, in particular, were used in this research: a strategy-focused sheet (parent A3) and an operational-focused sheet (child A3) [45]. The strategic A3 provides a framework for organizational priorities that foster the achievement of strategic planning goals and is linked to an operational A3, which is used as a tool to move a project forward.

Kaizen means a “change for the better” and thus translates continuous improvement into a problem-solving process [29]. This approach involves making small improvements and small changes within existing processes. A kaizen is prepared based on the goals of a strategic A3 report and is overseen by a management team member [43].

Control rooms, or obeya in Japanese, are places where team members can meet to assess current performance in a dynamic way and engage in discussion to support future performance [12,30,44]. Control rooms encourage cross-cutting management to help an organisation fully understand and grasp different organisational issues [13] and require strong collaboration between team members [14]. The goal of the strategic control room is to reflect the overall performance of an organization, and the control room is steered by the President and CEO and the Executive Committee [39,40]. The strategic control room is then broken down into tactical control rooms that are usually configured based on the departmental organizational structure. The tactical control room is overseen by a program director, such as the Director of Youth Programs. The tactical control room acts as a catalyst in the decision-making chain, as it allows for communication with the strategic control room and easily integrates with the operational control rooms. The operational control rooms allow managers to manage the daily operations that create value in the organization [39]. This tool tends to break down organizational barriers to allow staff to collaborate effectively during performance management meetings [43]. It also provides an opportunity for a team to share knowledge, build cohesion, and continuously improve [45].

The status sheet represents a standardised dialogue between an employee and his or her immediate superior. The sheet helps the employee learn about and better understand the organisation’s operations and prevent potential problems within a trajectory. A status sheet meeting is facilitated by a senior administrator from the management committee based on status sheets from the field.

Kata are small, structured practice routines. Learning and then combining these individual practice routines is a way of developing competency in the overall way or pattern of doing something [37]. It is important to emphasize that Kata should be considered not as a tool but rather as a state of mind that creates a context for how tools will be used [43].

**Figure 1:**
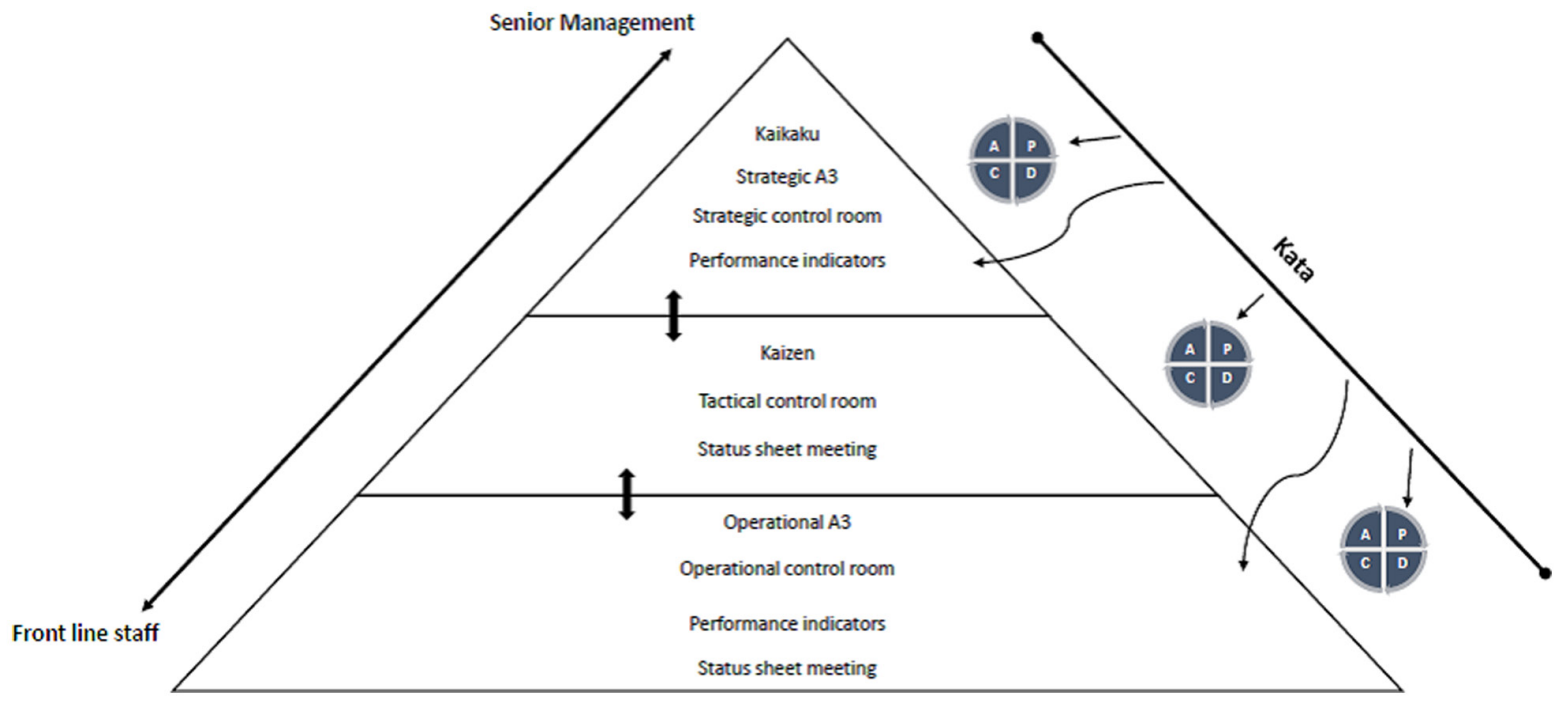
Links between different tools and PDCA cycles.

All of these tools use the Plan, Do, Check, Adjust (PDCA) problem-solving cycle popularised by Deming [16]. ***Figure 1*** synthesizes these various tools and how they are linked within the organization.

## Methods

This study uses action research, which consists of generating scientific knowledge to better understand and change the social reality of individuals [32]. This type of research has three distinguishing characteristics. First, it is done “with” people and not “on” people. Second, it focuses on action and the need for change rather than a methodological framework and becomes a starting point for cycles of adjustment. With this methodology, the researcher works closely with members of the system under study in a participatory process to solve a collective problem [17,18] and does so throughout a number of PDCA problem-solving cycles. Action research is used to support an organisational change strategy [19]. This methodology is based on pragmatist epistemology in management sciences [20].

This action research was carried out between June 2016 and October 2018. More than 350 hours of data collection was performed to create field notes taken from semi-structured interviews, focus groups and participant observation. Semi-structured interviews were conducted at the participants’ workplace with four psychosocial workers, six senior administrators, and five middle managers. Each interview lasted between 60 and 90 minutes and was conducted using a semi-structured interview guide. The questions aimed to ascertain what happened and why it happened (diagnosis) and what could have been done differently (reflection). Saturation was reached once the themes and categories became repetitive or no longer provided new or different information [34].

The data analysis strategies led to a detailed description of the various sources, including a full transcript of the content of all individual interviews from the audio recordings. These strategies related to grounded theory include three main coding steps: open coding, axial coding and selective coding [32].

Three 90-minutes focus groups were also conducted in October 2018 based on the organisation’s hierarchical logic, i.e., strategic, tactical and operational. The focus groups were largely based on the learning history approach [35]. A document of over 20 pages was developed from the individual interviews and given out to the focus groups that included people involved in the change effort and other work team members who could learn from this document.

Additionally, over 350 hours of data was recorded in field notes from non-participant observations during different interdisciplinary meetings and during strategic, tactical and operational steering-room activities. The field notes were helpful for recording relevant information throughout the data collection period and were also useful for interpreting observations [34].

The adopted methodology minimized the risks of bias thanks to in-depth knowledge of the study setting and great attention paid to the methodology employed [33]. To assess rigor in research, Guba and Lincoln [36] posit four criteria that are usually recognized for their application to quantitative research but that have been adapted to qualitative research. Criteria to assess the quality of positivist quantitative research (construct validity, internal validity, reliability and external validity) were adapted to the particular features of constructivist qualitative research using different adopted strategies (criteria related to confirmability, credibility, dependability and transferability). The data was validated through triangulation, which helped pinpoint the multiple perceptions and meanings of reality through the comments collected and limit potential sources of bias [32]. All qualitative data is stored in an NVivo database.

The research project was approved by the Research and Ethics Committee of the Université du Québec à Trois-Rivières (No. CER-18-244-07.01).

## Results

The results are presented based on the cyclical logic structured by the PDCA and action research methodology. ***Table 1*** presents a summary of the results. Each phase of the PDCA cycle touches upon the types of intervention used for the tools of the integrated performance management system and the results obtained for the service integration project. However, some phases may have overlapped due to the cyclical process of the research.

**Table 1:** PDCA cycle and resources used.


PDCA CYCLE	RESOURCES

**PLAN** 2016 Project management	Implementation of the integrated performance management system: kaikaku Strategic A3 sheet and A3 project: Goal of full integration

**DO** 2016–2017 Innovation	Leading of kaizen activities with operational teams One tactical control room for youth (liaison and coordination) Tracking of the A3 sheets in the strategic, tactical and operational control rooms New leaders with collaborative skills

**CHECK** 2017 Observations	Problems to measure Information flows in a top-down and bottom-up cascade Single tactical control room for both departments Control rooms that support integration processes Speed of action

**ADJUST** 2017–2018 Implementation of the status sheet	Implementation of the status sheet (liaison, coordination and full integration) Substantial gains to improve accessibility, continuity of services and workplace wellness Complementarity of control rooms New PDCA cycle

2018 to today From a project approach to the implementation of a continuous improvement culture	The different tools of the integrated performance management system are used in a complementary way


### Plan (PDCA)

In June 2016, senior managers invited 4 middle managers from the integrated youth teams and 4 senior managers to a kaikaku event and to an initial activity whose goal was to improve coordination between the youth programmes directorate and youth protection directorate. The meeting took place over two days.

At the start of the kaikaku, senior managers conveyed a very clear vision as part of a true client-centred strategy [21]:

- A child will never suffer alone.
- A child will always be supported in his or her pathway.
- Do the impossible to avoid putting the development or safety of a child in danger. (Children must receive the full range of services to ensure their development and safety.)

Internal partners (such as the intellectual disability directorate and the general social services directorate) and external partners were asked to contribute at a later date. As one staff member explained:

“Jimmy” is not just young; he has other needs too. He may have impairments and be autistic as well […].”

Prior to the kaikaku, the continuous improvement specialist documented the actual service pathway of a child and the child’s family. The initial service request was for a language impairment detected by a perinatal nurse when the child was 20 months old. The last intervention noted in the child’s record was a report that was under assessment, even though the child was almost 10 years old. First, the continuous improvement specialist documented response times, number of staff members, number of interventions, etc. The Chief Executive Officer (CEO) believes that it would have been useful to invite a child or their parents to participate in the service integration project. Nevertheless, two obstacles quickly became apparent. The first stemmed from the merger of 7 institutions and a new culture of collaboration that had to be created among different partners. The second related to the confidentiality of reported situations and the need to manage the risks of social stigmatization given the territory’s minimal client base (i.e., in a small community, everyone knows everyone else).

A major concern for the kaikaku participants was simplifying the child’s pathway through services. As youths in difficulty are truly at the heart of the integration project, the managers decided to give the project the name of a child— “Jimmy”—so that everyone saw this child as a real partner. The CEO stated that the kaikaku gave the project momentum:

“After doing the kaikaku, we made a radical change and stayed focused on our goal.”

The use of the A3 reports on the second day to deploy the integration project helped clarify the performance indicators that could improve service accessibility and continuity for the target clientele and each manager at the session signed it as a formal commitment to carry out and help implement the process. A member of the management team said:

“The A3 reports allowed people who were supposed to contribute to the project to reflect on the project [….] when the managers signed the collaboration contract at the beginning of the project […,] that’s when people really became engaged.”

### Do (PDCA)

Four kaizen workshops required more tactical and operational approaches and the idea was not to establish complete integration for each youth but rather to reflect on a trajectory that could be adjusted based on the profiles of each little “Jimmy” in the territory. The performance indicators were tracked in the tactical control room and in the multiple operational control rooms of the integrated teams.

Despite the A3 tracking in the strategic control room, the integration project encountered issues that seriously impeded the integration project. Difficulties arising from the leadership of the senior managers of the different youth directorates hindered cooperation between the two sectors involved in the integration project. According to Kodner and Kyriacou [46], weak integration at different organisational levels has an impact on integration at other levels. Another team member also spoke to this increased engagement:

“In some situations, we are still working in silos. We can’t work on Jimmy’s case on our own; these are young people with service episodes who may be transiently intellectually disabled or experiencing dependency […;] but all we have to help Jimmy together. The project helps us do that.”

### Check (PDCA)

The arrival, in 2018, of two new senior managers with backgrounds in collaborative practices allowed for a single tactical control room to be set up for these two directorates. This was a major innovation. A middle manager had this to say about this innovation:

“Implementing the tactical room jointly between the youth program and the youth protection directorate has advantages, such as continuity. The tactical control room mobilized everyone in a new integration context.”

The tactical control room is where the continuum of services required by “Jimmy” and any areas of discontinuity identified by the operational control rooms are monitored. The comments of a middle manager support the importance of the tactical control room:

“We have concrete results […;] we’re taking action and things are happening […;] there’s follow-up in the other control rooms.”

Improvement Kata are an important practice methodology used during activities at operational or visual stations that identify improvement opportunities and next steps based on a PDCA cycle.

An example of cooperation and a staff member’s concern for service integration was made through an Improvement Kata in the operational control room. Lack of contact over a long period led to dissatisfaction among foster families who need ongoing support to help a foster child who is coping with personal and family issues. With this approach, the institution can now confirm that all children placed with foster families are met with monthly. “Jimmy” no longer suffers alone and is always supported. The target was then set by the integrated youth team.

### Adjust (PDCA)

Given the slow progress in the integration project and the lack of results to help Jimmy due to a lack of leadership, in June 2017 the CEO proposed creating a status sheet to better understand and document the trajectory through dialogue between an employee and his or her immediate superior. This new step created an anchor point for a new PDCA cycle. This individual status sheet was added as a complement to the activities in the operational control rooms and aimed to resolve the problems of accessibility, continuity and workplace well-being. Managers received training on the status sheets. Discussions were held to link the status sheets with the goals set out within the strategic A3 reports and the A3 projects. To reinforce this new practice, the CEO relied on the relationship skills of the continuous improvement specialist. The coaching of managers and teams was one reason for Project Jimmy’s success.

Within six months, the status sheet helped minimise problems tracking significant indicators meant to improve the targets set at the start of the integration project. This tool opened the door to service integration by getting the other youth directorates involved to alleviate problems related to fragmentation that, in the words of a middle manager, “could let a Jimmy fall through the cracks.” One manager was impressed by the speed of this tool and said:

“For me, the status sheet was a revelation out of the whole process.”

Once a week, all managers are invited to a conference call with the associate chief executive officer to report on each team’s progress. Called “status sheet meetings,” these weekly calls held every Friday between 8:00 a.m. and 9:00 a.m. let staff assess performance in terms of service accessibility and continuity in the trajectory and report on progress made and obstacles encountered since the previous meeting. For example, one question asked at the meeting relates to the number of users who do not show up for their appointments. For each no-show, the staff member is asked to explain why. Another issue relates more to staff well-being: the manager asks the staff member about their perception of their workload, and if the staff member feels overwhelmed, action is taken immediately. Comments from senior manager highlight the potential of this tool of the integrated performance management system:

“Without the status sheet, we would have no links between our control rooms; there would be a piece missing. The status sheet meetings allow staff to see how proactively removing obstacles to operational performance puts “Jimmy” at the heart of this approach.”

Six months after the status sheet was implemented in complementarity with the control rooms, improvements had been made in relation to targets to enhance service accessibility and continuity. At the start of the integration project, the average time between the assessment and a first intervention was 16.96 days, whereas the ministerial target was 12 days. In November 2017, the average time had decreased to 8.35 days. This significant time reduction helps staff respond more quickly to protect the needs of vulnerable children and prevent their situations from deteriorating. The service intensity, which had been 0.96 interventions per day per worker, improved significantly and rose to 3.6 interventions per day per worker. A 275% improvement over two years indicates improved access to services. Improving service intensity in turn optimizes staff case load and reduces the wait times for families experiencing problems. Clear improvements were also noted in the implementation of intervention plans, for which the ministerial target was 85%, as the results showed an increase in intervention plans from 70% to 85% in a statutory context and from 10% to 43% in a voluntary context. The fact that families can have an intervention plan makes it easier to determine the problem and the best tools to resolve it, which in turn creates better linkage, better coordination, and fuller integration. Finally, the disability insurance rate dropped from 14.30% to 8.70%, which generated substantial savings. When staff return to work, this provides better service continuity for families by ensuring that their services do not become fragmented. In addition to indicator performance, team actions helped fulfill the vision set out during the kaikaku, i.e., that Jimmy will never be alone again.

Communication is a major challenge in an organisational integration project [23]. The deployment of the operational control rooms and the status sheet are a determining factor in the integration of services and the functioning of the integrated youth teams. A staff member and a middle manager said:

“There are advantages […] in our day-to-day reality […,] as problems crop up […] and then solutions do too.”

The advantage of using the weekly status sheet to improve “Jimmy’s” service trajectory is that it generates quick wins in terms of improved coordination and improved communication at all organisational levels.

## Discussion

The goal of this exploratory qualitative study is to explain whether integration is supported by an integrated performance management system, particularly through the deployment of control rooms and the status sheet. At IHSSC, the issue of integration is being addressed through the implementation of a service trajectory that ensures service accessibility and continuity for children, youth and family clients and that improves the well-being of staff to reduce the risk of early attrition.

This research supports the idea that the integrated performance management system is a collaborative model for trajectory management and that it can therefore support vertical and horizontal integration [1,11,24]. Significant benefits from collaboration include the sharing of interdisciplinary expertise, improved collective decision-making through idea sharing, and an individual and collective ability to improve indicators to meet targets. Activities in control rooms have had a real impact on the development of interprofessional collaboration and a better coordination. The approach used demonstrates the importance of Leutz’s idea [27] that integration can only occur when all stakeholders are involved in its planning and implementation. The trajectory-based approach draws from the principle of user value [24]. “Jimmy” became a strong symbol for this integration project.

To improve the use of the strategic control room, it would have been interesting to have visual boards of “Jimmy’s” trajectory, with the idea that visual management provides a better understanding of processes, highlights opportunities for improvement, and reduces waste [25]. Visual boards for trajectories would be in line with the idea that visual management removes organisational silos by creating cross-cutting management to help everyone involved become aware of and grasp different organisational issues [13]. These research findings also confirm that horizontal integration is challenging, and teams can be slow to see progress [7,11]. The main difficulties in this case arose from human issues [26] that hindered collaboration with other directorates that nevertheless also provide clinical or advisory services in “Jimmy’s” trajectory. One principle described by Leutz [27] is that integrating services, or even simply collaborating, requires managers to broaden their knowledge, perspectives and interests. These observations suggest that the logic of interdepartmental competition prevented staff from reflecting on how to collaborate better together.

The central component of this integration project has been the implementation of an integrated youth team through strategic, tactical and operational control rooms to offer coordinated services. This action research shows how interdependence was created between the two directorates in particular through the tactical and operational control room thanks to trajectory review meetings that fostered discussions between each directorate’s respective stakeholders and forced them to broaden their knowledge, perspectives and interests. The control rooms and the status sheet became true catalysts for integration. These complementary tools fostered the linkage, coordination and full integration of services for the benefit of children, youth and families. The innovation of deploying a single tactical control room integrating the youth protection directorate and the youth programmes directorate supports better communication and, accordingly, confirms that communication among all stakeholders is a key issue in integration processes [23].

The control rooms and the status sheet reinforced the project proposed by the CEO in 2016 to integrate services for the well-being of the territory’s children, youth and families, which in turn reinforces the idea of complementarity between vertical and horizontal integration [6,7]. For the youth programmes directorate and youth protection directorate at the IHSSC, these two tools of the integrated performance management system seem to be turning into formal collaboration mechanisms that are building trust through better knowledge of the integrated team members. These tools are also formalising relationships between different staff members to promote greater fluidity in “Jimmy’s” pathway by standardising certain practices to increase the reliability of organisational performance data.

The integrated performance management system deployed for the Jimmy project upholds the idea that performance is multidimensional and is contingent on the balanced approach of the quadruple aim model. The various tools resulting from the management model has generated not only financial gains but also gains in the areas of accessibility, continuity and staff engagement. Although lacking at the start of the project, performance measurement saw great progress, as the status sheet consolidated the processes defining the trajectory’s measurable objectives and therefore collected performance data generated by the integration project.

## Lessons Learned

- Importance of defining the concept of integration during the kaikaku to give the project meaning. The stakeholders of the trajectory must have a better knowledge of an integration continuum.
- Personalizing the project by naming it “Jimmy.” He became the catalyst for the integration project. He participates in the creation of meaning. Each worker who took part in the project had a very clear reference to a “Jimmy” in a vulnerable situation in his entourage. The speakers were keen to contribute to Jimmy’s well-being in a more fluid and improved trajectory. There was no fear or resistance to integrating the services.
- The use of the tools of the integrated performance management system supports the stakeholders on a daily basis. Staff members no longer face the challenges of working with vulnerable clients alone, as they are met with on a weekly basis both through the status sheet and the control room. These tools of the integrated performance management system support active listening and create the conditions for requests to be grouped so that staff can adequately respond to children’s needs through a standardisation of formal communications.
- The integrated performance management system is first and foremost a collaborative management model supporting the integration and improvement of care and services. The control rooms and the status report demonstrate the proactive action of operational teams in identifying obstacles and solutions to an optimal path to meet customer needs.
- The creation of a single tactical control room between two complementary departments that supports processes to better coordinate services. Observations from this research suggest that the tactical control room makes any obstacles in the trajectory flow instantly visible to managers. Having staff members participate in the operational control room helps them work together to ensure service access and continuity while drawing from expertise from different areas.

## Conclusion and Limitations of the Study

Like many institutions in Quebec’s health and social services network, the IHSSC had to improve its performance while tackling the integration challenges posed by the 2015 reform. To face this challenge, the institution decided to deploy an integrated performance management system. Supported by the different tools of the integrated performance management system, the “Jimmy” integration project reinforces the implementation of linkage, coordination and full integration models that become strong connections between teams. The status sheet and control room are tools that vertically integrate different hierarchical levels and horizontally integrate various front-line stakeholders through the user-oriented trajectory.

Based on this study’s observations, future research avenues could address how an integration project supported by different tools of the integrated performance management system improves employee workplace well-being to ensure accessible and continuous services. Future research could also determine how target indicators change over a longer period and explore the optimal conditions to ensure the organisational sustainability of this type of integration project for different clienteles. Overall, it would be useful to look at the principles of the “learning organization” as part of an organizational transformation that fosters service integration processes. Useful outcomes could also come about through a convergence of the integrated performance management system with organizational capacity to improve results in a context in which practice innovation is encouraged.
